# Resistance remains a problem in treatment failure

**DOI:** 10.7448/IAS.17.4.19756

**Published:** 2014-11-02

**Authors:** Martin Obermeier, Robert Ehret, Andreas Wienbreyer, Hauke Walter, Thomas Berg, Axel Baumgarten

**Affiliations:** 1 Laboratory, Medical Center for Infectious Diseases, Berlin, Germany; 2 Clinical Research, Medical Center for Infectious Diseases, Berlin, Germany; 3 Outpatients Clinic, Medical Center for Infectious Diseases, Berlin, Germany

## Abstract

**Introduction:**

Proven resistance against HIV drugs, either by phenotyping or genotyping is a rare event in clinical trials. The overall assumption of drug resistance disappearing is additionally driven by the recommendations to screen for transmitted drug resistance, leading to large numbers of examinations with relatively low rates of resistance. Goal of our analysis was to assess if drug resistance in treatment failure is also decreasing outside of clinical trials.

**Materials and Methods:**

The MIB database at timepoint of analysis consists of data from 2876 HIV infected patients. Besides various laboratory parameters, clinical data and treatment history is included. HIV-1 protease and reverse transcriptase sequences were analyzed using the HIV-GRADE drug resistance algorithm. As in only a small number of patients genotypic resistance testing for integrase inhibitors was performed, mainly due to reimbursement reasons, it was assumed that failing treatment on a previous integrase inhibitor containing regimen is equate to resistance.

**Results:**

Of the 2876 patients in the database, 220 had a treatment change due to treatment failure between 2009 and 2012, a genotypic resistance testing at an appropriate timepoint of maximum four weeks before treatment change and a treatment duration of at least six months before treatment failure. In 2009, 61% of patients showed no drug resistance while 39% showed resistance against one or more drug classes (two or more drug classes: 19.5%; three or more drug classes: 2.4%, four drug classes 2.4%). In 2012, no resistance was found in 52% of patients while resistance against three or more drug classes was found in nearly 14% of patients (one or more: 48%; two or more 23%; four classes: 4.5%).

**Conclusions:**

Treatment failure with viral load sufficiently high for drug resistance testing was not frequently observed in our database. Nevertheless, treatment failure was often associated with drug resistance against at least one drug class. With more use of the newer drug classes, resistance against those new classes will become more common and rates of multiclass resistance will be increasing.

